# Single VHH-directed BCMA CAR-NK cells for multiple myeloma

**DOI:** 10.1186/s40164-023-00461-8

**Published:** 2023-11-27

**Authors:** Quan Ren, Yingling Zu, Hongchang Su, Qiumei Lu, Bin Xiang, Yanping Luo, Jishuai Zhang, Yongping Song

**Affiliations:** 1 Department of Hematology, The Affiliated Cancer Hospital of Zhengzhou University and Henan Cancer Hospital, Zhengzhou, Henan, 450008 China; 2 Shenzhen Pregene Biopharma Company Ltd, Shenzhen, 518118 China; 3 Department of Hematology, The First Affiliated Hospital of Zhengzhou University, Zhengzhou, Henan, 450052 China

**Keywords:** BCMA, CAR-NK, Multiple Myeloma, VHH

## Abstract

Natural killer (NK) cells are promising alternatives for the production of “off-the-shelf” CAR products, posing a lower risk of cytokine release syndrome (CRS) than CAR-T cells. We synthesized four single VHH-directed anti-BCMA CARs, incorporating various intracellular regions (2B4 *versus* CD28) and hinge domains (CD28 *versus* IgG1) and ectopically producing IL-15. NK cells derived from peripheral blood (PB) were expanded ex vivo by K562-mbIL21 feeder cells. Stable CAR transduction was obtained through lentiviral transduction with the BaEV-Rless pseudotyped lentiviral vector. BCMA-CD28-IL15 CAR-NK cells with ectopic expression of IL-15 exhibited superior cytotoxicity were compared to BCMA-CD28 CAR-NK cells lacking IL-15 and BCMA-hIgG1-IL15 CAR-NK cells with an IgG1 hinge domain. We further assessed the cytotoxic capabilities of BCMA-2B4-IL15 CAR-NK cells with 2B4 intracellular domain. The BCMA-CD28-IL15 CAR-NK cells revealed stronger cytotoxicity and higher cytokine secretion against BCMA^+^ tumor cells than BCMA-2B4-IL15 CAR-NK cells in vitro. In the MM.1S-Luc mouse model, BCMA-CD28-IL15 CAR-NK inhibited the growth of tumor cells and prolonged mouse survival. These results show that the single VHH-directed BCMA CAR-NK cells exhibited remarkable specific killing ability, making them a potential candidate for immunotherapy in multiple myeloma treatment.

**Supplementary Information:**

The online version contains supplementary material available at 10.1186/s40164-023-00461-8.

**To the Editor:**

Natural killer (NK) cells are promising alternatives for the production of “off-the-shelf” CAR products, posing a lower risk of cytokine release syndrome (CRS) than CAR-T cells [1, 2]. Nanobodies (Nbs), or “single domain antibodies” (sdAbs) or “variable domains of heavy chain of heavy-chain antibodies” (VHHs), are naturally occurring antibodies that lack light chains in Camelidae and shark species peripheral blood [3]. Our previous studies have confirmed that PRG1801 CAR-T cells using a single VHH targeting one BCMA epitope are effective in the clinical treatment of multiple myeloma (MM) [4]. In this study, we prepared single VHH-directed anti-BCMA CAR-NK cells and evaluated their cytotoxic properties.

First, we confirmed our optimized protocol for ex vivo expansion of NK cells derived from peripheral blood (PB). Using K562-mIL-21 as feeder cells can make NK cells rapidly expand and obtain high-purity NK cells [5, 6] (Supplemental methods and data). The NK cells achieved approximate 4,000-fold expansion with a final purity of more than 90%. We used BaEV-Rless envelope pseudotyped lentiviral vector, which can bind to the amino-acid-transporter receptors ASCT1 and ASCT2 that are highly expressed on activated NK cells [5, 7], in the lentiviral package system. Stable CAR transduction efficiency was obtained.

We then selected MM.1S, Daudi, NCI-H929, and RS4;11 cell lines with different BCMA expression levels as target cells for in vitro cytotoxicity validation (Supplementary Fig. S2D). The CAR ectopically produced IL-15 allowing NK cells to prolong in vivo proliferation [8, 9]. We encoded the IL-15 gene in the CAR construct and verified the functional impact of IL-15 expression. A BCMA-CD28-IL15 CAR with ectopic IL-15 expression and a BCMA-CD28 CAR without IL-15 were synthesized (Fig. 1A). There was a statistically significant difference in the cytotoxic activity of BCMA-CD28-IL15 CAR-NK cells toward MM.1S and Daudi cells, compared to BCMA-CD28 CAR-NK and NK cells (*P* < 0.0001, n = 3) (Fig. 1D). Furthermore, the secretion levels of IFN-γ, granzyme B, and IL-15 were increased in the BCMA-CD28-IL15 CAR-NK cells (*P* < 0.0001, n = 3) (Fig. 1E).

**Fig. 1:**
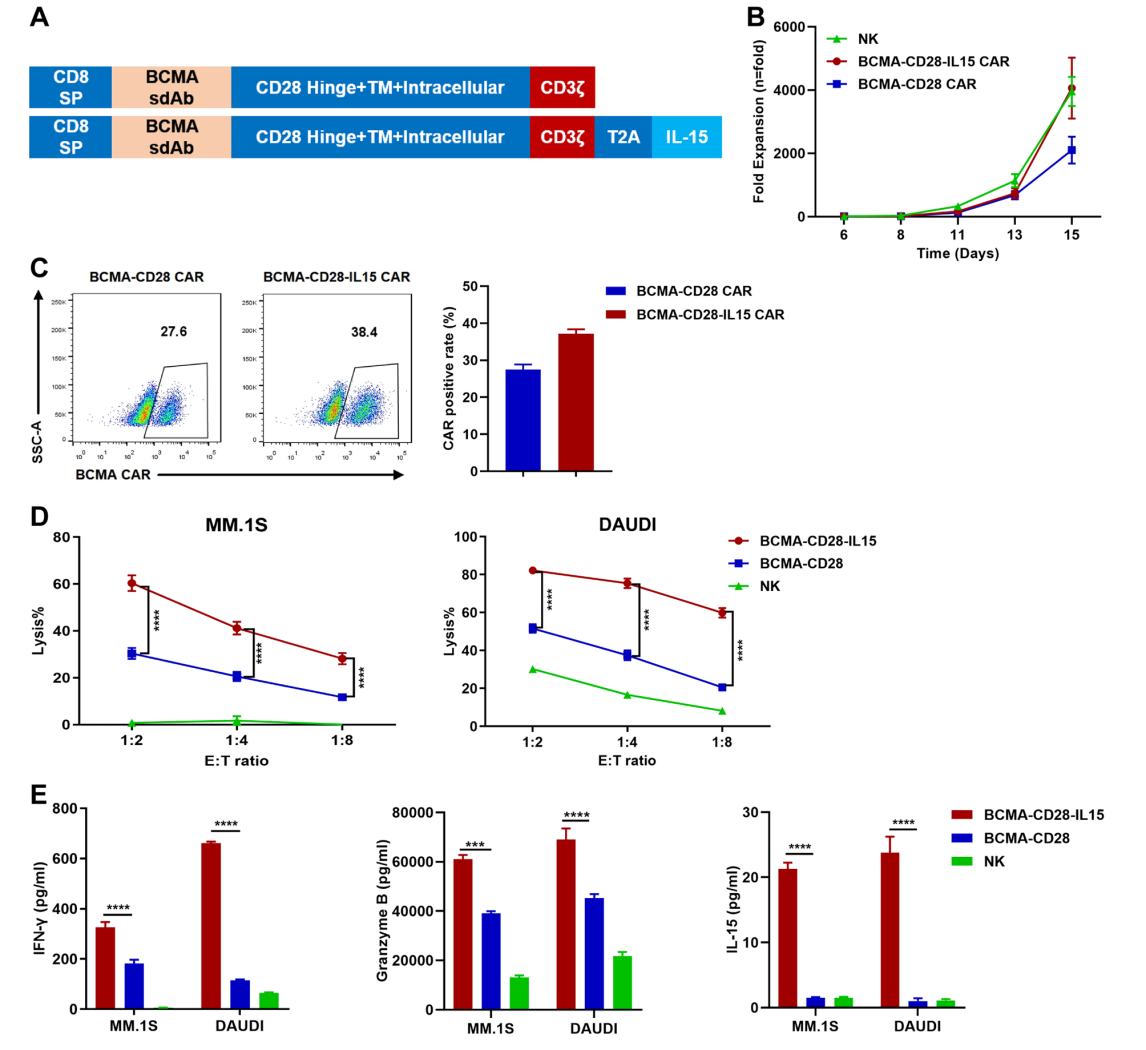
BCMA-CD28-IL15-engineered NK cells showed enhanced antitumor activity compared to BCMA-CD28 CAR-NK cells. **(A)** Schematic diagrams of BCMA CAR-NK constructs. **(B)** The fold expansion curve of NK cells, BCMA-CD28-IL15 CAR-NK cells, and BCMA-CD28 CAR-NK cells. **(C)** BCMA CAR expression of NK cells on day 14. **(D)** The cytotoxic activity of BCMA-CD28-IL15 CAR-NK cells vs. BCMA-CD28 CAR-NK cells and ex vivo-expanded NK cells against MM.1S and Daudi cells using a lactate dehydrogenase release assay (n = 3; ****, *P* < 0.0001). The numbers of effector cells were calculated as CAR-positive cells. NK cells were used to adjust the different CAR-positive cells. **(E)** MM.1S and Daudi cells were cocultured with BCMA-CD28-IL15 CAR-NK cells, BCMA-CD28 CAR-NK cells, or NK cells at an E:T ratio of 1:2 for 24 h. IFN-γ, granzyme B, and IL-15 secretion in supernatants was measured by ELISA (n = 3; **, *P* < 0.01; ***, *P* < 0.001; ****, *P* < 0.0001)

Subsequently, a BCMA-hIgG1-IL15 CAR with an immunoglobulin G-based (IgG1) hinge was synthesized to verify the functional impact of different hinge regions. After coculture with MM.1S cells for 16 h, the BCMA-CD28-IL15 CAR-NK cells exhibited superior cytotoxic lysis over BCMA-hIgG1-IL15 CAR-NK cells at all tested E:T ratios (*P* < 0.0001, n = 3). The BCMA-CD28-IL15 CAR-NK cells secreted significantly more IFN-γ against MM.1S cells than BCMA-hIgG1-IL15 CAR-NK cells (*P* < 0.0001, n = 3) (Supplementary Fig. S1).

The optimal intracellular domain in CAR-NK is not fully understood [10]. A BCMA-2B4-IL15 CAR with co-stimulator 2B4 was also constructed for in vitro functional comparison. 2B4 is an NK cell-specific receptor. CAR constructs with 2B4 costimulatory domains have shown superior antitumor efficacy [11, 12]. The fold expansion and CAR-positive rate of the BCMA-2B4-IL15 CAR-NK cells and BCMA-CD28-IL15 CAR-NK cells are shown in Supplementary Figure S2B, C. The BCMA-CD28-IL15 CAR-NK cells demonstrated higher levels of cell lysis than the BCMA-2B4-IL15 CAR-NK cells against MM.1S and NCI-H929 cells (*P* < 0.01, n = 3). However, in terms of antitumor activity in the Daudi and RS4;11 cells, no significant differences were observed. Both CAR structures showed minor cytotoxic effects against BCMA-negative RS4;11 cells (Supplementary Fig. S2E). The secretion levels of IFN-γ, granzyme B, and IL-15 by BCMA-CD28-IL15 CAR-NK cells, with the exception of the RS4;11 cells, were higher than those in the BCMA-2B4-IL15 CAR-NK cells with the indicated cell lines (*P* < 0.05, n = 3) (Supplementary Fig. 2F).

Finally, we assessed the in vivo antitumor activity of the BCMA-CD28-IL15 CAR-NK cells in the immunocompromised NCG mouse model (Fig. 2A). The MM.1S-luc cell injection was defined as day 0, and BCMA-CD28-IL15 CAR-NK cells were injected nine days after tumor inoculation. After BCMA-CD28-IL15 CAR-NK cells administration, the BCMA-CD28-IL15 CAR-NK group had a lower bioluminescence intensity, compared with the control or mock-NK groups (*P* < 0.0001, n = 4) (Fig. 2B, C), which demonstrated the ability to inhibit tumor cells in vivo. We further found that the mice in the BCMA-CD28-IL15 CAR-NK group maintained their weight throughout the observation period, while the mice in both the control and mock-NK groups, losing large amounts of weight from day 27 until death, reflecting the degree of disease progression (Fig. 2D). The BCMA-CD28-IL15 CAR-NK group had significantly prolonged survival compared with the control and mock-NK groups (*P* < 0.05, n = 4) (Fig. 2E). The median survival time of the BCMA-CD28-IL15 CAR-NK group was undefined until being euthanized on day 65. The median survival time was 42 days for the control group and 40 days for the mock-NK group. There was no significant difference in survival between the control and mock-NK groups (*P* = 0.8355, n = 3). However, a limitation of the mouse model is that we did not include the BCMA-2B4-IL15 CAR-NK group.

**Fig. 2:**
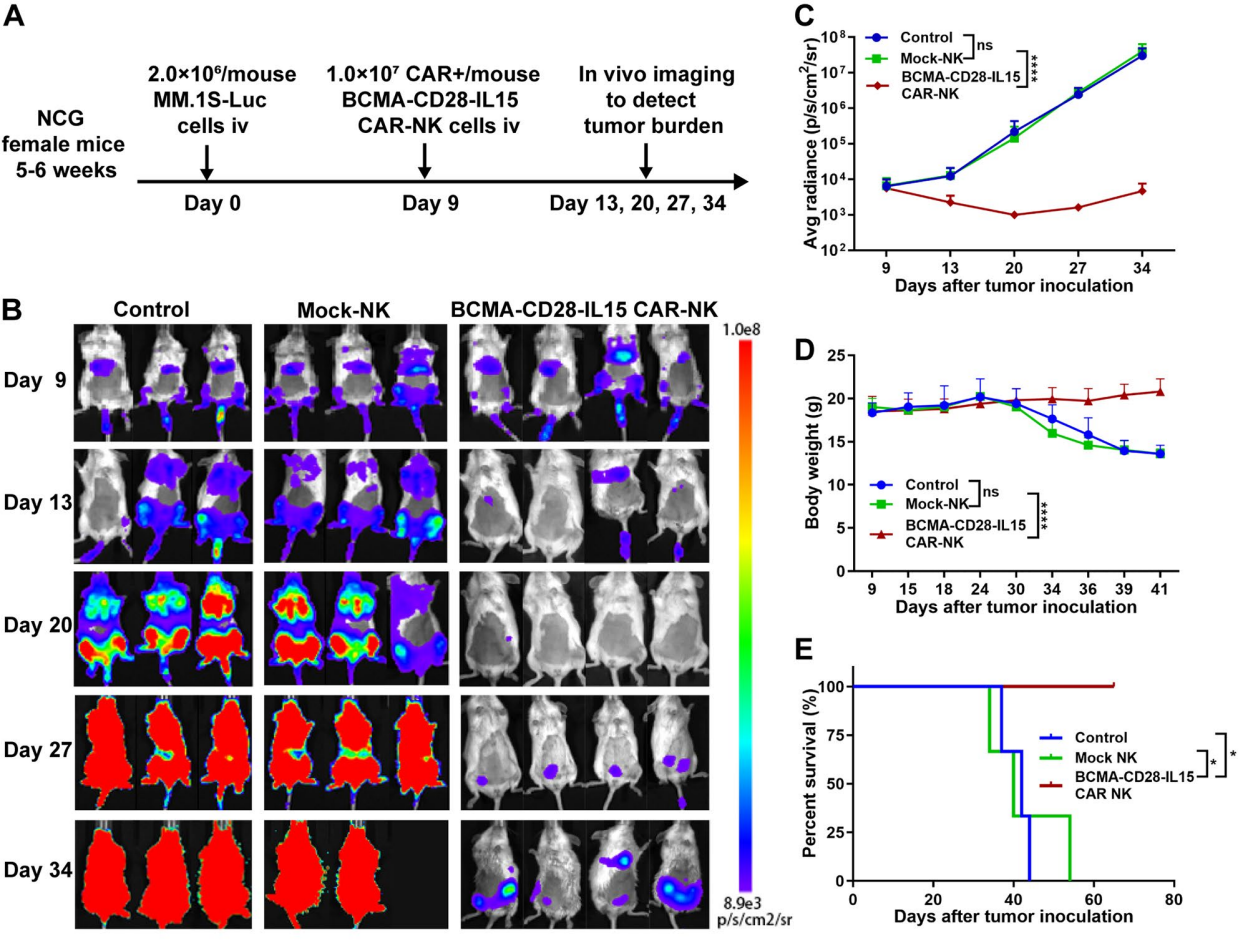
In vivo antitumor activity of BCMA-CD28-IL15 CAR-NK cells in the MM.1S-Luc transplanted NCG mouse model. **(A)** Schematic diagram of the mouse in vivo study. The NCG mice received an *IV* injection of 2.0 × 10^6^ MM.1S-Luc cells on day 0. Nine days after tumor inoculation, the mice were randomly divided into three groups (n = 3 for the control and mock-NK groups; n = 4 for the BCMA-CD28-IL15 CAR-NK group) according to the average radiance of the bioluminescence imaging. The mice were intravenously administered cryoprotectant (a solvent control), mock-NK cells, and BCMA-CD28-IL15 CAR-NK cells on day 9. **(B)** Bioluminescence images on days 9, 13, 20, 27, and 34. **(C)** Statistical analysis of the bioluminescence intensity on different days (****, *P* < 0.001; ns, no significance). **(D)** Body weight of each group measured on different days (****, *P* < 0.0001; ns, no significance). **(E)** Kaplan–Meier survival curves of mice in vivo. A statistical analysis of survival between groups was performed using the log-rank test. Statistical significance in survival rates was obtained after Bonferroni correction for multiple comparisons (*, *P* < 0.05)

In conclusion, the single VHH-directed BCMA CAR-NK cells exhibited remarkable specific killing ability, making them a potential candidate for immunotherapy for MM.

### Electronic supplementary material

Below is the link to the electronic supplementary material.

Supplementary Material 1

## Data Availability

The data that support the findings of this study are available from the corresponding author upon reasonable request.

## Abbreviations

CAR: Chimeric antigen receptor
BCMA: B-cell maturation antigen
CRS: Cytokine release syndrome
VHH: Variable domain of heavy chain of heavy-chain antibody
PB: Peripheral blood
BaEV: Baboon retrovirus envelope pseudotyped lentivectors
