# Supplementary material for: Single VHH-directed BCMA CAR-NK cells for multiple myeloma
Source: Exp Hematol Oncol. 2023 Nov 27;12:98. doi: 10.1186/s40164-023-00461-8 (PMC10680242; doi:10.1186/s40164-023-00461-8)
Supplement: Supplementary file 1 — Supplementary Material 1 [file 40164_2023_461_MOESM1_ESM.docx]

**Supplementary Material**

**Materials and methods**

**Cell lines and culture conditions**

K562-based feeder cells expressing membrane-bound IL-21 (mbIL21) and CD137 ligand were constructed by lentiviral transduction for NK cell expansion. Daudi, MM.1S, NCI-H929 and RS4;11 cell lines were purchased from ATCC and cultured in RPMI 1640 (HyClone, USA) media supplemented with 10% fetal bovine serum (TransGen, China). The 293TS lentiviral vector packaging cell line (derived from HEK293T cells, ATCC) was cultured in SMM293TⅡ and OPM293-CD05 media (Opmbiosciences, China).

**Plasmid construction and lentiviral production**

The antigen recognition domain of this BCMA binding VHH sequence from an alpaca antibody phage display library was previously described[1]. We cloned the CAR gene into a lentivirus transfer plasmid (Pre-SIN, Pregene Biopharma, China) under the control of a human EF-1α promoter. The CAR construct sequences included the CD8 signaling peptide, BCMA VHH sequence, CD28 or IgG1 hinge region, CD28 transmembrane region, CD28 or 2B4 intracellular costimulatory molecule, intracellular signaling molecule CD3ζ and human IL-15 gene. All CAR sequences were synthesized by Igebio (Guangzhou, China). We used a third-generation lentiviral system consisting of three helper vectors. The lentiviral vectors carrying the CAR genes were packaged with transfer plasmid, packaging plasmid (pMDLg/pRRE and pRSV-Rev), and envelope plasmid (pCMV-BaEV-Rless) and transfected into 293TS cells by using polyethyleneimine (PEI, Polysciences, Warrington, Pennsylvania, USA). After transfection for 48 h, cell supernatants were collected and filtered through a 0.45-µm filter and then concentrated by ultracentrifugation at 10,000 × g for 2 h at 4°C. The lentiviral supernatants were stored in 0.9% NaCl with human albumin (CSL Behring, USA) as a stabilizer at -80°C and used directly or thawed immediately before transduction.

**Generation of CAR-modified NK cells**

Peripheral blood mononuclear cells (PBMCs) were collected from healthy donors and isolated by a density gradient technique with Ficoll (Sigma‒Aldrich, USA). All healthy donors provided written informed consent according to the protocol approved by the Local Ethics Committee of the Affiliated Tumor Hospital of Zhengzhou University. CD56^+^ NK cells were purified with CD56 Microbeads (Miltenyi, Germany). Then, CD56^+^ NK cells were activated with K562-mbIL21 feeder cells and cultivated in NK MACS GMP Medium (Miltenyi, Germany) supplemented with 3% autologous plasma and 100 IU/ml recombinant human IL-2 (Beijing Sihuan Biopharmaceutical, China) at a density of 2.5×10^5^ NK cells/ml on day 0. On day 4, the activated NK cells were centrifuged and exchanged for a double volume of fresh media. Two days later (Day 6), 2×10^6^ NK cells were transduced with lentiviral vectors encoding CAR constructs at a multiplicity of infection (MOI) of 10 on Retronectin (TaKaRa Bio Inc., Japan)-coated 6-well plates (2 ml final volume in each well). RetroNectin was coated in nontreated 6-well plates either overnight at 4°C or for 2 h at 37°C prior to use. After 48 h, NK cells were stimulated again with feeder cells at a density of 1.25×10^5^ cells/ml. The NK cells were expanded and propagated until harvest.

**Flow cytometry analysis**

PE-conjugated CD56 and FITC-conjugated CD3 antibodies (BioLegend, San Diego, CA, USA) were used to label primary NK cells and T cells. BCMA CAR surface expression on transduced NK cells was detected by staining with a BCMA-His tag (FITC, ARCO, Beijing, China). The BCMA expression levels on cell lines were measured by using PE-labeled anti-BCMA antibodies (Bio Legend, USA). All samples were washed twice and resuspended in 300 µL staining buffer before quantitative analysis by flow cytometry, while dead cells were excluded by 7AAD staining (Thermo Fisher, USA). FACS analysis was performed on a FACS Calibur flow cytometer (BD Bioscience, USA) and analyzed by FlowJo 7.6.1 software.

**Cytotoxicity assay of CAR-NK cells *in vitro***

To assess the *in vitro* cytotoxicity of CAR-NK cells against tumor cells, we performed a lactate dehydrogenase (LDH) release assay. Each target cell line at a dose of 3×10^4^ cells was cocultured with effector cells (CAR-NK or untransfected NK) at effector-to-target (E:T) ratios of 1:2, 1:4 and 1:8 in a 96-well round-bottom plate for 16 h. The numbers of effector cells were calculated as CAR-positive cells. NK cells were used to adjust the different CAR-positive cells. Then, 50 µL of the supernatant from each well was collected and transferred to a new 96-well plate, followed by an equal volume of lactate dehydrogenase (LDH) assay buffer. After incubation for 30 minutes at room temperature in the dark, the experiment was conducted as specified by the manufacturer using the CytoTox 96® Non-Radioactive Cytotoxicity Assay Kit (Promega, USA). The percentage of specific lysis was calculated by the following formula: $\frac{Experimental- Effector Spontaneous- Target Spontaneous}{Target Maximum- Target Spontaneous} \times100.$

**Cytokine measurements**

Cytokines, including interferon-gamma (IFN-γ), granzyme B, and IL-15, in the supernatants collected from cocultures after 24 hours of incubation with an E:T ratio of 1:2 were measured by enzyme-linked immunosorbent assay (ELISA; Dakewei, China) according to the manufacturer’s instructions. The results for each cytokine were calculated in pg/mL based on the standard concentration and analyzed for significant differences between different groups.

**Mouse model and *in vivo* assessment**

Six- to seven-week-old female NCG (NOD/ShiLtJGpt-*Prkdc*^em26Cd52^*Il2rg*^em26Cd22^/Gpt, Gempharmatech, Jiangsu, China) were injected with MM.1S-Luc cells (2.0×10^6^/mouse) through the tail vein to establish the mouse model. Nine days after tumor inoculation, the mice were randomized into three treatment groups according to the average radiance of the bioluminescence imaging. Three mice were included in the control and mock-NK groups, and 4 mice were included in the BCMA-CD28-IL15 CAR-NK group. Then, the mice were intravenously administered cryoprotectant (same volume of NK cells as a solvent control), mock-NK cells (same number of NK cells as the BCMA-CD28-IL15 CAR-NK group) and BCMA-CD28-IL15 CAR-NK cells (1.0×10^7^ CAR^+^NK/mice). Luminescence analysis was performed using an in vivo bioluminescence imaging system (IVIS, PerkinElmer, USA) on day 9 (before CAR-NK administration), 13, 20, 27 and 34 to monitor tumor growth or eradication by BCMA-CD28-IL15 CAR-NK cells. All mice were weighed every 2 days. Animal experiments were approved by the Animal Care and Use Committee of ANLING Laboratories (Shenzhen, China).

**Statistical analysis**

Statistical analysis was performed with GraphPad Prism 9.0 software (GraphPad Software, USA). Unpaired Student’s t test (two-tailed) was performed for comparisons between two groups, one-way ANOVA for multiple comparisons and survival curves were performed using the Kaplan–Meier method, and differences of statistical analyses were compared with the log-rank test. Data are presented as the mean ± SD. A *P* value< 0.05 was considered statistically significant.

**Supplementary Figures**

**
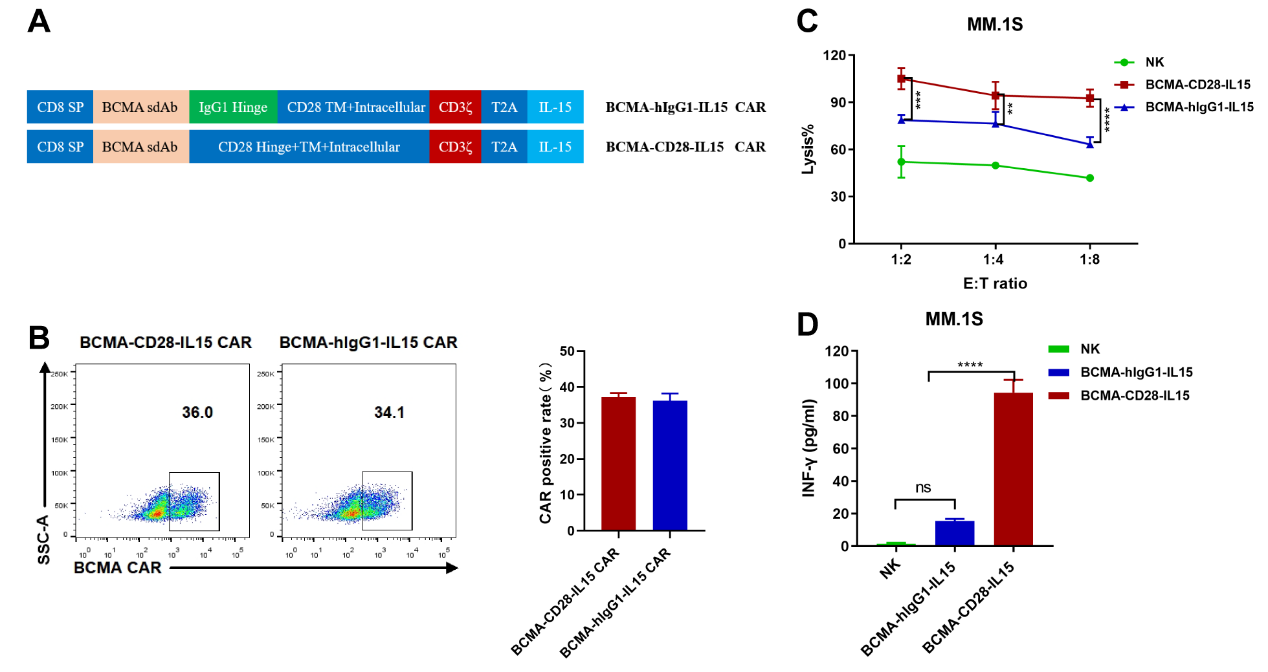
Fig. S1** Comparison of anti-BCMA CAR-NK cells with the hinge region of IgG1 (BCMA-hIgG1-IL15 CAR) or CD28 (BCMA-CD28-IL15 CAR). **(A)** Schematic diagrams of BCMA CAR-NK constructs with different IgG1 or CD28 hinge domains. **(B)** The expression of BCMA-CD28-IL15 CAR and BCMA-hIgG1-IL15 CAR on NK cells. **(C)** The cytotoxic activity of BCMA-CD28-IL15 CAR-NK cells *vs.* BCMA-hIgG1-IL15 CAR-NK cells and ex vivo-expanded mock-NK cells against MM.1S cells using a lactate dehydrogenase release assay (n= 3; **, *P* < 0.01; ***, *P* < 0.001; ****, *P* < 0.0001). **(D)** MM.1S cells were cocultured with BCMA-CD28-IL15 CAR-NK cells, BCMA-hIgG1-IL15 CAR-NK cells or NK cells at an E:T ratio of 1:2 for 16 h. Supernatants were harvested for measurement of IFN-γ secretion by ELISA (n=3; ****, *P* < 0.0001; ns, no significance).

**
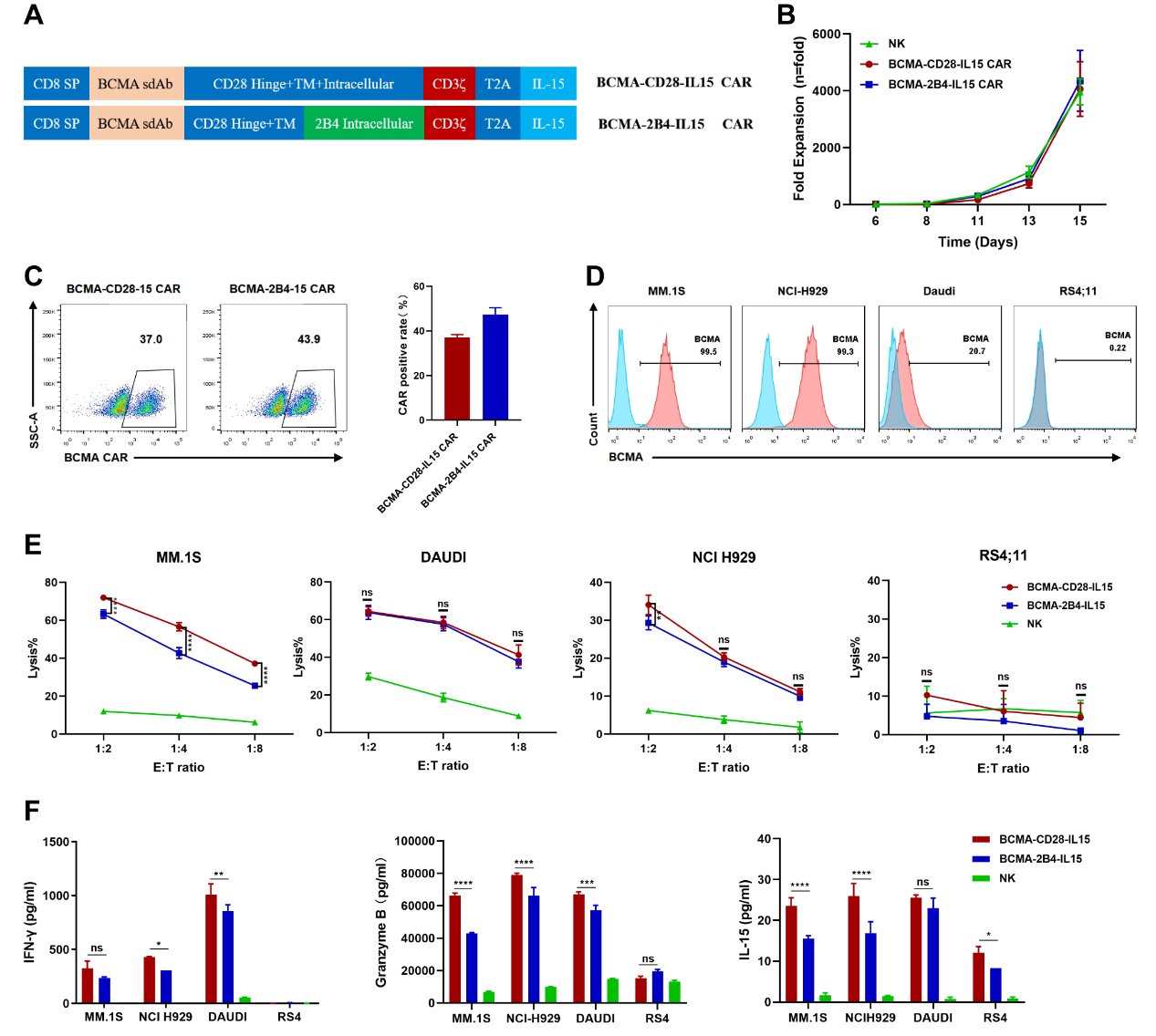
Fig. S2** BCMA-CD28-IL15 CAR-NK cells revealed stronger cytotoxicity and cytokine release levels than BCMA-2B4-IL15 CAR-NK cells in vitro. **(A)** Schematic diagrams of BCMA CAR-NK constructs with different intracellular activation domains, CD28 *vs.* 2B4. **(B)** The fold expansion curve of mock-NK, BCMA-CD28-IL15 CAR-NK and BCMA-2B4-IL15 CAR-NK cells. **(C)** The expression of BCMA-CD28-IL15 CAR and BCMA-2B4-IL15 CAR on NK cells was detected by flow cytometric analysis on day14. **(D)** The surface expression of BCMA on the cancer cell lines. Representative results are shown. **(E)** Cytotoxicity assay of BCMA CAR-NK cells in vitro. BCMA CAR-NK cells and NK cells were cocultured with the indicated cell lines at E:T ratios of 1:2, 1:4 and 1:8 for 16 h before the lactate dehydrogenase release assay (n=3; **, *P* < 0.01; ****, *P* < 0.0001; ns, no significance). **(F)** The indicated cells were cocultured with BCMA-CD28-IL15 CAR-NK cells, BCMA-2B4-IL15 CAR-NK cells or mock-NK cells at an E:T ratio of 1:2 for 24 h. Supernatants were harvested for measurement of IFN-γ, granzyme B, and IL-15 secretion by ELISA (n= 3; *, *P* < 0.05; **, *P* < 0.01; ***, *P* < 0.001; ****, *P* < 0.0001; ns, no significance).

**Reference**

1. Han L, Zhang J-S, Zhou J, Zhou K-S, Xu B-L, Li L-L, et al. Single VHH-directed BCMA CAR-T cells cause remission of relapsed/refractory multiple myeloma. Leukemia. 2021;35(10):3002-6.
